# JAK Inhibitors in the Treatment of T-Cell Lymphomas: Current Evidence and Future Directions

**DOI:** 10.3390/cancers18050799

**Published:** 2026-02-28

**Authors:** Gardenia Taza, Naveed Ahmed, John L. Vaughn

**Affiliations:** Department of Medicine, NYU Grossman Long Island School of Medicine, Mineola, NY 11501, USA; gardenia.taza@nyulangone.org (G.T.); naveed.ahmed@nyulangone.org (N.A.)

**Keywords:** Janus kinase inhibitors, T-cell lymphoma, peripheral T-cell lymphoma, cutaneous T-cell lymphoma

## Abstract

T-cell lymphomas are a heterogeneous group of lymphoid neoplasms with a variable prognosis. They can be further divided into cutaneous T-cell lymphomas and peripheral T-cell lymphomas. Treatment options are relatively limited for patients with relapsed or refractory disease. Janus kinase (JAK) inhibitors have emerged as promising new drugs for these lymphomas, as increasing evidence supports the JAK and signal transducer and activator of transcription (STAT) pathway as a potential target. The objective of this review is to summarize the current evidence supporting the use of JAK inhibitors in the treatment of T-cell lymphomas and highlight areas for future research. Although many JAK inhibitors have been developed for the treatment of autoimmune conditions, only a subset of these have been tested in T-cell lymphomas and reported in the literature. These include abrocitinib, cerdulatinib, golidocitinib, ruxolitinib, tofacitinib, and upadacitinib. Other drugs are currently being tested in clinicals trials, including pacritinib and ivarmacitinib, but results are not yet available. Most of the published data are for ruxolitinib, which was found to have a clinical benefit rate of up to 53% in patients with PTCL with activating JAK and/or STAT mutations. Response durations are limited, which may be overcome through combination therapies in the future. JAK inhibitors are associated with multiple adverse effects, including cytopenias and infections, and long-term safety data are lacking for newer agents. Future studies will need to clarify long-term safety and efficacy through well-designed clinical trials involving larger groups of patients.

## Simple Summary

T-cell lymphomas are cancers that originate from white blood cells called T-cell lymphocytes. These cancers can grow slowly or aggressively, and they can affect any part of the body, including the skin and lymph nodes. New treatments are needed for patients with T-cell lymphomas that have become resistant to treatment. Drugs called Janus kinase (JAK) inhibitors may be an effective treatment option for these patients. The first commercially available JAK inhibitor, ruxolitinib, was found to be safe and effective in early-phase clinical trials. Additional JAK inhibitors are currently in clinical development for patients with T-cell lymphomas. Future studies will be needed to determine how well these drugs work in larger groups of patients and to clarify any long-term adverse effects. The purpose of this review is to provide an overview of these medications in the treatment of T-cell lymphomas.

## 1. Introduction

T-cell lymphomas (TCLs) represent a large heterogeneous group of lymphoid malignancies originating from T cells or natural killer (NK) cells. The recent fifth edition (2022) of the World Health Organization (WHO-5) recognizes nine families of mature T-cell and NK-cell neoplasms as well as a separate family of precursor T-lymphoblastic neoplasms [1]. In the literature, TCLs are often broadly categorized as either cutaneous T-cell lymphomas (CTCLs), which primarily affect the skin, or peripheral T-cell lymphomas (PTCLs), which include nodal or extranodal subtypes. Among CTCLs, mycosis fungoides (MF) accounts for approximately 50% of cases, while the most common PTCL subtypes include peripheral T-cell lymphoma, not otherwise specified (PTCL-NOS), angioimmunoblastic T-cell lymphoma (AITL), and anaplastic lymphoma kinase (ALK)-positive and ALK-negative anaplastic large cell lymphoma (ALK-positive and ALK-negative ALCL) [2].

Despite their heterogeneity, TCLs are generally more challenging to treat than their B-cell counterparts. Prognosis varies widely across subtypes, from indolent CTCLs to aggressive PTCLs, such as monomorphic epitheliotropic intestinal T-cell lymphoma, which has a median survival of only 7.8 months [3]. Janus kinase (JAK) inhibitors have emerged as promising therapeutic agents, as increasing evidence highlights the JAK/STAT (Janus kinase and signal transducer and activator of transcription) pathway as a potential target in various T-cell lymphomas. Activating mutations of STAT5B and STAT3 were found in γδ-T or NK-cell-derived lymphoma cells, and treatment with a JAK inhibitor caused dose-dependent inhibition of cell growth [4]. In CTCL, point mutations have been detected in JAK1 (0.9%), JAK3 (2.7%), STAT3 (0.9%), and STAT5B (3.6%) [5,6], with JAK3 mutations associated with advanced stage disease and large-cell transformation [6].

Activation of the JAK/STAT pathway has also been observed in other T-cell lymphoproliferative disorders [7,8,9]. In a cohort of 98 cases of PTCL-NOS, 26 exhibited phosphorylated STAT6 (p-STAT6), which was positively correlated with Ki67 expression (*p* = 0.012) and expression of p52 (*p* < 0.001). Notably, p-STAT6 expression was related to shorter survival (*p* = 0.008) [10]. Phosphorylated STAT 3 (p-STAT3) was also associated with decreased overall survival (OS). pSTAT3 was seen within 27% of PTCL-NOS, 29% of angioimmunoblastic T-cell lymphoma (AITL), 93% in ALK-positive ALCL, and 57% in ALK-negative ALCL [11]. Activating mutations in JAK1, JAK2, STAT3, or STAT5B have been noted in 40% of cases of T-cell large granular lymphocytic leukemia (T-LGL) and 75% of T-cell prolymphocytic leukemia [7,9].

JAK inhibitors bind to the enzymatic site of Janus kinases, thereby inhibiting STAT protein phosphorylation and disrupting downstream cytokine-driven signaling pathways [12]. JAK inhibitors are widely used for the treatment of inflammatory diseases such as rheumatoid arthritis or ulcerative colitis [13]. Some JAK inhibitors have been approved for the treatment of myelofibrosis and polycythemia vera [14]. However, there are no US Federal Drug Administration (FDA)-approved JAK inhibitors for T-cell lymphomas. To date, ruxolitinib is the sole agent approved for the treatment of chronic graft-versus-host disease (cGVHD), a post-stem-cell transplant complication that may arise in some individuals with lymphoma [15]. The aim of this review is to provide an overview of JAK inhibitors in the management of T-cell lymphomas. We will discuss six JAK inhibitors, including preliminary efficacy and notable adverse effects, omitting some inhibitors such as baricitinib due to lack of data in this patient population.

## 2. Ruxolitinib

Ruxolitinib is a selective JAK1 and JAK2 inhibitor. It is available in both topical and oral formulations. Its oral formulation is currently approved for the treatment of steroid-refractory acute graft-versus-host disease (aGVHD) and chronic graft-versus-host disease (cGVHD) in adults and pediatric patients 12 years and older [16,17]. aGVHD and cGVHD are potential complications in T-cell lymphoma patients who have undergone allogeneic hematopoietic cell transplantation (HCT) [18,19]. For aGVHD, doses start at 5 mg twice daily, and for cGVHD, doses start at 10 mg twice daily.

Ruxolitinib has been used to achieve disease control and to bridge to allogeneic HCT, leading to a durable remission [20]. Jaramillo et al. describe a patient with cutaneous relapsed T-cell acute lymphoblastic leukemia (T-ALL) with JAK3 mutations following an allogeneic blood cell transplant [21]. Given major contraindications to tofacitinib (JAK1, JAK2, JAK3 inhibitor), combination therapy with tofacitinib and ruxolitinib was not pursued. Instead, salvage therapy with ruxolitinib monotherapy showed complete remission of skin lesions at day 50. Ruxolitinib was then discontinued after 5 months due to cutaneous relapse [21]. Cao et al. describe a patient with chronic diarrhea, found to have indolent T-cell lymphoma of the gastrointestinal tract (ITCL-GI) with a STAT3::JAK2 fusion. His diarrhea resolved after 3 months of ruxolitinib 10 mg twice daily. Despite his clinical improvement, repeat endoscopy did not show any changes in histomorphologic changes [22].

A retrospective review of 6 patients treated with ruxolitinib (4 oral and 2 topical) found a clinical response in 3 cases. A patient with CTCL treated with oral ruxolitinib had a complete response for 3 years, while another patient with subcutaneous panniculitis-like T-cell lymphoma (SPTCL) with a JAK2 point mutation showed diminishing lesions for a year. Plaque thinning was seen in a patient with MF on topical ruxolitinib. Notably, oral ruxolitinib doses varied across patients from 5 mg twice daily to 20 mg twice daily [23]. Levy et al. reported a case of a 16-year-old male with relapsing hemophagocytic lymphohistiocytosis and SPTCL who achieved remission in 4 months with ruxolitinib 15 mg twice daily. Ruxolitinib was self-tapered and discontinued by the patient, with disease relapse noted 8 months after discontinuation. Ruxolitinib was then re-initiated with eventual complete remission [24].

In a phase 2 biomarker-driven study, 45 patients with PTCL and 7 patients with MF received ruxolitinib 20 mg twice daily on a 28-day cycle. Patients were placed in one of three cohorts: (1) patients whose tumors had activating JAK and/or STAT mutations; (2) patients whose tumors lacked mutations but exhibited ≥30% pSTAT3 expression by immunohistochemistry; or (3) patients whose tumors did not meet either criterion. Clinical benefit response (CBR) was defined as complete response (CR), partial response (PR), or stable disease for 6 months. The CBR of PTCL and CTCL in cohorts 1, 2, and 3 were 48%, 36%, and 18%, respectively. Only 1 of 7 MF patients achieved PR, and notably, their tumor only exhibited 20% of pSTAT3 expression. A total of 8 patients (15% of cases) achieved responses of greater than 1 year, including 4 of 5 T-LGL, 1 PTCL-NOS, 1 with AITL, 1 with primary cutaneous ALCL, and 1 with MF. This study suggests that in PTCL and non-MF CTCL, JAK/STAT mutations and/or pSTAT3 expression in ≥30% of tumor cells predicted CBR to ruxolitinib (*p* = 0.02) [25].

## 3. Tofacitinib

Tofacitinib, a JAK1, JAK2, and JAK3 inhibitor, is currently approved for select autoimmune conditions such as rheumatoid arthritis, psoriatic arthritis, and ulcerative colitis, among others. Tofacitinib has been increasingly utilized off-label in the treatment of various dermatological conditions, including cutaneous manifestations of autoimmune conditions [26]. Studies investigating the effectiveness of tofacitinib in T-cell lymphomas remain largely preclinical. In one study, tofacitinib was shown to inhibit tumor growth in EBV-associated T-cell lymphoma cells [27].

Suhl et al. reported successful treatment of a patient with cutaneous sarcoidosis and Sézary syndrome using a combination of tofacitinib and romidepsin [28]. Romidepsin is a histone deacetylase inhibitor with FDA approval for CTCL. Although romidepsin monotherapy initially led to improvement, the patient’s condition plateaued after one month. The addition of tofacitinib resulted in reduced generalized erythema, minimal skin tenderness, and the resolution of axillary and inguinal lymphadenopathy [25]. A treatment regimen of tofacitinib and ruxolitinib was also successful in a patient with T-PLL with relapsed disease 16 months after undergoing a stem cell transplant. Targeted next-generation sequencing identified two missense JAK3 mutations. In vitro studies showed that the combination of tofacitinib and ruxolitinib exhibited a synergistic effect in leukemic cells. This patient was initially started on tofacitinib 5 mg twice daily, further increased to 10 mg twice daily after 15 days of treatment. Ruxolitinib was added one month after initiating tofacitinib, and within the first two months of dual therapy, there was an improvement in fatigue, low-grade fevers, night sweats, and a regression of leukemia cutis lesions [29].

## 4. Golidocitinib

Golidocitinib is a selective JAK1 inhibitor currently under active investigation specifically for the treatment of PTC [30]. The rationale for targeting JAK1 alone is that selective JAK1 inhibition may result in lower toxicity [31].

JACKPOT8 part B, a multinational phase 2 single-arm trial, is currently the largest active trial at the time of this review’s writing [31]. Patients ≥ 18 years of age with relapsed/refractory PTCL who had received at least 1 prior line of therapy and had an Eastern Cooperative Oncology Group (ECOG) performance status of 0–2 met the inclusion criteria. Patients received oral golidocitinib 150 gm once daily until disease progression or other discontinuation criteria were met. A total of 104 patients were enrolled between 26 February 2021 and 12 October 2022. Median follow-up was 13.3 months. As of the most recent update in January of 2024, the objective response rate was 44.3%, with 24% of patients achieving CR and 21% of patients achieving PR. The primary grade 3 and 4 adverse effects observed have been neutropenia and thrombocytopenia. To date, 1 event of death has been recorded secondary to fungal pneumonia thought to be secondary to golidocitinib-induced immunosuppression [31].

Golidocitinib received conditional approval in China based on the results of JACKPOT8. The full therapeutic potential of golidocitinib continues to be assessed in ongoing studies. A phase 2 study (NCT05963347) is evaluating the combination of golidocitinib with CHOP (cyclophosphamide, doxorubicin, vincristine, and prednisolone) in newly diagnosed PTCL. A separate clinical trial (NCT06733051) for extranodal Natural Killer/T-cell lymphoma is exploring the efficacy of golidocitinib and benmelstobart, an anti-PD-L1 inhibitor, in patients with relapsed or refractory disease.

## 5. Cerdulatinib

Cerdulatinib is an experimental dual inhibitor of spleen tyrosine kinase (SYK), JAK1, JAK2, and JAK3. It is being widely tested in hematological malignancies and dermatological conditions. A preclinical study in adult T-cell leukemia/lymphoma found that cerdulatinib suppressed cell proliferation and cell viability more than pan-JAK or SYK-selective inhibitors alone. Furthermore, murine models treated with cerdulatinib resulted in lower tumor burden [32].

An expansion phase II study of 98 patients with refractory PTCL or CTCL treated with cerdulatinib 30 mg orally twice daily noted good tolerability and favorable responses. An interim analysis demonstrated an ORR of 35% in CTCL patients [33,34]. Responses in MF and SS varied greatly, with an ORR of 45% and an ORR of 17%, respectively. No patients with SS achieved a complete response, whereas 9% of MF patients did. However, in CTCL patients, rapid improvement in pruritus was observed, independent of tumor response. At study conclusion, the ORR was 51.9% in the angioimmunoblastic T-cell lymphoma and T follicular helper cell lymphoma (AITL/TFH) subgroup, 31.8% for other histological subtypes, and a surprising 0% for PTCL-NOS. In the AITL/TFH subgroup, complete remission was achieved in 10 patients and partial remission in 4 patients. These responses were generally maintained for more than 6 months, with five patients showing persistent responses for more than 12 months. Adverse effects included lipase and amylase increases (with no clinical pancreatitis), diarrhea, neutropenia, anemia, and fatigue, all of which were treated by standard care [33,34].

## 6. Upadacitinib

Upadacitinib is a selective JAK1 inhibitor with FDA-approval for a range of chronic inflammatory diseases. Upadacitinib has been used off-label for CTCL in select case studies.

Castillo et al. describe a case of erythrodermic mycosis fungoides treated successfully with upadacitinib. Initial skin biopsies were inconclusive, and in the absence of significant lymphadenopathy, a diagnosis of atopic dermatitis was made. The patient was started on upadacitinib 15 mg daily. After 2 months, repeat biopsies confirmed MF stage 3. By week 16, the patient achieved complete response with less than 10% of body surface area affected [35]. Another case of MF misdiagnosed as atopic dermatitis found notable improvement in both skin lesions and pruritus within two weeks of initiating upadacitinib [36].

## 7. Abrocitinib

Abrocitinib is a selective JAK1 inhibitor approved for moderate to severe atopic dermatitis. It is currently being investigated for other dermatological, allergic, and inflammatory conditions. There is only one case noting abrocitinib in combination with interferon and phototherapy to treat a refractory case of recalcitrant folliculotropic MF. The initiation of abrocitinib led to significant improvement in pruritus within four weeks, and a noticeable reduction in plaques and papules was observed after three months [37].

## 8. Safety of JAK Inhibitors

In clinical trials, JAK inhibitors were associated with multiple adverse events. In patients with PTCL and MF who were treated with ruxolitinib, common adverse events (occurring in greater than 5% of patients) were anemia, neutropenia, thrombocytopenia, diarrhea, fatigue, and febrile neutropenia [25]. Golidocitinib was associated with neutropenia, thrombocytopenia, increased transaminases, pneumonia, and leukopenia. For cerdulatinib, the most common treatment-emergent adverse events were increased amylase, diarrhea, nausea, increased lipase, and fatigue [34]. A list of adverse effects associated with JAK inhibitors is shown in Table 1. In these trials, adverse events requiring discontinuation of the treatments were uncommon. For example, in the JACKPOT8 trial, only 9% of patients treated with golidocitinib discontinued the drug due to treatment-emergent adverse events [31].

In 2021, the FDA published a warning that JAK inhibitors might be linked to a higher incidence of lymphomas and other malignancies [42]. This warning was based on the ORAL surveillance study, which compared the risk of cardiac events, malignancy, and infection in patients with rheumatoid arthritis receiving tofacitinib with those receiving a tumor necrosis factor inhibitor [43]. Since other JAK inhibitors work through similar pathways, the warning was extended to the entire drug class [44]. Several cases have been reported in the literature, as described below [45,46,47,48,49,50].

Cohen et al. described two cases of severe CTCL relapse in patients treated with ruxolitinib for cGVHD after allogeneic HCT. Both individuals presented with relapsed MF, notable for erythroderma and extensive skin tumors, with blood involvement in one case [45].

Knapp et al. reported a case of refractory erythema elevatum diutinum treated with tofacitinib. The patient ultimately developed lymphomatoid papulosis (LyP) with ocular involvement after 8 weeks of treatment [46].

Linuma et al. described a case of LyP in a patient treated with upadacitinib for rheumatoid arthritis. Her presentation was notable for red-to-brown papules on her extremities two weeks after upadacitinib initiation. The authors suggested that the immunosuppression from JAK1 inhibition increased her susceptibility to LyP. Her lesions improved with upadacitinib discontinuation and topical corticosteroids [47].

Mo et al. reported a patient with severe atopic dermatitis, who was started on upadacitinib with partial relief of his symptoms. His course was complicated by multiple episodes of bullous impetigo, leading to discontinuation of upadacitinib and rapid deterioration of his symptoms. One month after upadacitinib discontinuation, the patient underwent a cheek biopsy that revealed pilotropic MF [48].

Lamolet et al. described a patient with early MF who was misdiagnosed as atopic dermatitis and treated with upadacitinib and baricitinib (another JAK1/JAK2 inhibitor that has not been studied in lymphoma patients). Despite an initial brief improvement in symptoms, the patient was noted to have worsening skin lesions and the development of inguinal lymphadenopathy, which the authors attributed to JAK inhibitor use [49]. A similar transient response with eventual worsening disease was also noted by Martin-Torregrosa et al. in a patient treated with upadacitinib [50].

In 2025, a retrospective cohort study identified cases of CTCL following exposure to dupilumab (anti-IL-4Rα), tralokinumab (anti- IL-13), or JAK inhibitors. They identified no patients diagnosed with CTCL following treatment with JAK inhibitors alone or tralokinumab alone [51]. It remains unclear whether JAK inhibitors cause secondary malignancies (e.g., through their immunosuppressive effects) or if the observed associations are due to confounding.

## 9. Conclusions and Future Directions

T-cell lymphomas remain challenging diseases to treat due to relatively limited treatment options, especially in the relapsed/refractory setting [52]. Relapsed/refractory T-cell lymphomas are associated with poor outcomes, emphasizing the need for novel therapies. Activation of the JAK/STAT pathway has emerged as a driver in the pathogenesis of multiple T-cell lymphoma subtypes, providing a compelling rationale for JAK inhibitor use. However, more data from clinical trials are needed in order to support their use as standard therapies. Table 2 shows a list of ongoing clinical trials that are testing JAK inhibitors in the treatment of T-cell lymphomas. Many of the trials involve the use of combination therapies, including the use of JAK inhibitors in the frontline setting. Ruxolitinib and golidocitinib are both being tested in separate trials in combination with cyclophosphamide, doxorubicin, vincristine, and prednisone (CHOP) for newly diagnosed peripheral T-cell lymphomas.

Among the commercially available JAK inhibitors, ruxolitinib has the most published data for the treatment of T-cell lymphomas. Ruxolitinib has demonstrated favorable responses across both CTCL and PTCL, especially in patients with activating JAK/STAT mutation or elevated pSTAT3 expression [25]. However, response durations have been limited. Combination with BCL-2 or PI3K inhibitors highlights a potential strategy to overcome possible resistance mechanisms [53]. Another area for future research will be to determine whether one type of JAK inhibitor is preferred over another for certain T-cell lymphoma subtypes.

In addition to promoting objective disease responses, JAK inhibitors may also have a role in the amelioration of symptoms. JAK inhibitors improve symptoms, including erythema, pruritus, and cytopenias. The use of oral JAK inhibitors for symptomatic improvement alone may be a particularly attractive option for patients with indolent T-cell lymphomas such as mycosis fungoides, perhaps in combination with topical JAK inhibitors [54]. Additional clinical trials using standardized quality of life measures will help support the role of JAK inhibitors for these diseases.

The FDA has raised concerns regarding the safety of JAK inhibitors and the potential increased risk of cancer in patients who receive these medications for autoimmune diseases. However, a causal association between JAK inhibitors and malignancy has not been established. Autoimmune disease itself is associated with an increased risk of lymphoma, representing a possible source of confounding. A recent retrospective study did not find any cases of CTCL in treatment with JAK inhibitors alone, contradicting other case reports [51].

In conclusion, JAK inhibitors are generally well-tolerated drugs that may offer meaningful clinical responses, either through disease control or symptomatic improvement in patients with T-cell lymphomas. These drugs are being further tested as part of combination therapies, including in patients with newly diagnosed T-cell lymphomas. Further research should focus on patient selection through biomarker-driven approaches and combination strategies to enhance therapeutic efficacy and durability.

## Figures and Tables

**Table 1:** Adverse effects of JAK inhibitors.

Drug	Adverse Effects
Ruxolitinib	Infection, thrombocytopenia, anemia, neutropenia, bruising, dizziness, headache [38].
Tofacitinib	Infection, GI perforation, rheumatoid and psoriatic arthritis, ulcerative colitis, anemia, neutropenia, elevated liver enzymes, elevated lipids, lymphoproliferative disorders [39].
Golidocitinib	Infection, thrombocytopenia, anemia, neutropenia, hypertriglyceridemia, hyperuricemia, elevated liver enzymes, pyrexia [31].
Cerdulatinib	Infection, anemia, neutropenia, elevated lipase and amylase, fatigue [33].
Upadacitinib	Infection, GI perforation, anemia, neutropenia, hyperlipidemia, elevated liver enzymes, rheumatoid arthritis, psoriatic arthritis, ankylosing spondylitis, ulcerative colitis [40].
Abrocitinib	Infection, cardiovascular events, thrombosis, thrombocytopenia, lymphopenia, lymphoma, lung cancer [41].

**Table 2:** Clinical trials of JAK inhibitors for T-cell lymphomas.

ClinicalTrials.gov ID	Drug	Disease	Phase	Anticipated Completion Date
NCT05010005	Ruxolitinib Duvelisib	R/R T or NK cell lymphoma	I	August 2027
NCT02974647	Ruxolitinib	R/R T or NK cell lymphoma	II	November 2025
NCT07283822	Ruxolitinib Pembrolizumab	R/R T-cell lymphoma	II	January 2029
NCT07278856	Ruxolitinib CHOP	Newly diagnosed nodal T-follicular helper cell lymphoma	I	31 March 2027
NCT05745714	Ruxolitinib Venetoclax Dexamethasone Cyclophosphamide Cytarabine	R/R precursor T-lymphoblastic lymphoma/leukemia	I/II	2 February 2032
NCT06698822	Tofacitinib (Topical)	CTCL	II	19 October 2026
NCT07279584	Golidocitinib	R/R PTCL	II	31 December 2027
NCT07032532	Golidocitinib	Newly diagnosed PTCL	II	December 2028
NCT06757387	Golidocitinib Chidamide	R/R PTCL	I/II	30 December 2030
NCT07209163	Golidocitinib Linperlisib Tazemetostat	R/R PTCL	I/II	March 2027
NCT06739265	Golidocitinib CHOP	Newly diagnosed PTCL	I/II	1 September 2026
NCT05963347	Golidocitinib CHOP	Newly diagnosed PTCL	II	31 July 2026
NCT06733051	Golidocitinib Benmelstobart	R/R extranodal NK/T-cell lymphoma	II	December 2028
NCT07093710	Golidocitinib Mitoxantrone GemOx	R/R PTCL	I/II	1 August 2029
NCT06630091	Golidocitinib CHOP	Newly diagnosed PTCL	II	5 July 2029
NCT07234162	Golidocitinib Chidamide Pralatrexate Gemcitabine Belinostat	R/R PTCL	III	31 October 2028
NCT06701344	Golidocitinib CHOP	Intestinal T-cell lymphoma	II	4 December 2027
NCT07081607	Golidocitinib Azacitadine Chimamide	PTCL	II	16 July 2028
NCT06573138	Golidocitinib Anti PD-1 antibody Selinexor	R/R NK/T-cell lymphoma	II	1 July 2027
NCT06966154	Golidocitinib Tislelizumab Selinexor	R/R NK/T-cell lymphoma	I/II	30 May 2028
NCT07300514	Golidocitinib	PTCL	III	30 December 2029
NCT06855823	Golidocitinib Pomalidomide	R/R PTCL	I/II	December 2026
NCT04858256	Pacritinib	R/R T-cell lymphoma	II	November 2028
NCT05900089	Ivarmacitinib	R/R NK/T-cell lymphoma	I	1 June 2026
NCT06519526	Ivarmacitinib	R/R PTCL	II	31 August 2027

CHOP: cyclophosphamide, doxorubicin, vincristine, and prednisone, CTCL: Cutaneous T-cell lymphoma, NK: Natural killer, PTCL: Peripheral T-cell lymphoma, R/R: Relapsed/refractory.

## Data Availability

No new data were created or analyzed in this study.

## Abbreviations

The following abbreviations are used in this manuscript:

aGVHD	Acute graft-versus-host disease
cGVHD	Chronic graft-versus-host disease
CHOP	Cyclophosphamide, doxorubicin, vincristine, and prednisone
CTCL	Cutaneous T-cell lymphoma
FDA	Food and Drug Administration
HCT	Hematopoietic cell transplantation
JAK	Janus kinase
MF	Mycosis fungoides
NK	Natural Killer
PTCL	Peripheral T-cell lymphoma
STAT	Signal transducer and activator of transcription
T-ALL	T-cell acute lymphoblastic leukemia
